# Genome-wide identification and expression profiling of invertase gene family for abiotic stresses tolerance in *Poncirus trifoliata*

**DOI:** 10.1186/s12870-021-03337-3

**Published:** 2021-11-25

**Authors:** Bachar Dahro, Yue Wang, Ahmed Alhag, Chunlong Li, Dayong Guo, Ji-Hong Liu

**Affiliations:** 1 Key Laboratory of Horticultural Plant Biology (MOE), College of Horticulture and Forestry Science, Huazhong Agricultural University, Wuhan, 430070 China; 2 Department of Horticulture, Faculty of Agriculture, Tishreen University, Lattakia, Syria

**Keywords:** *P. trifoliata*, Invertase, Genome-wide identification, Sucrose hydrolysis, Gene expression, Abiotic stresses

## Abstract

**Background:**

Sucrose (Suc) hydrolysis is directly associated with plants tolerance to multiple abiotic stresses. Invertase (INV) enzymes irreversibly catalyze Suc degradation to produce glucose (Glc) and fructose (Frc). However, genome-wide identification and function of individual members of the *INV* gene family in *Poncirus trifoliata* or its *Citrus* relatives in response to abiotic stresses are not fully understood.

**Results:**

In this report, fourteen non-redundant *PtrINV* family members were identified in *P. trifoliata* including seven alkaline/neutral INV genes (*PtrA/NINV1–7*), two vacuolar INV genes (*PtrVINV1–2*), and five cell wall INV isoforms (*PtrCWINV1–5*). A comprehensive analysis based on the biochemical characteristics, the chromosomal location, the exon–intron structures and the evolutionary relationships demonstrated the conservation and the divergence of *PtrINV*s. In addition, expression analysis of *INV* genes during several abiotic stresses in various tissues indicated the central role of *A/NINV7* among *INV* family members in response to abiotic stresses. Furthermore, our data demonstrated that high accumulation of Suc, Glc, Frc and total sugar contents were directly correlated with the elevated activities of soluble INV enzymes in the cold-tolerant *P. trifoliata*, *C. ichangensis* and *C. sinensis*, demonstrating the potential role of soluble INV enzymes for the cold tolerance of *Citrus*.

**Conclusions:**

This work offered a framework for understanding the physiological role of *INV* genes and laid a foundation for future functional studies of these genes in response to abiotic stresses.

**Supplementary Information:**

The online version contains supplementary material available at 10.1186/s12870-021-03337-3.

## Background

Abiotic stresses including cold, salinity, and drought  cause cellular osmotic stress leading to cell membrane damage and protein structure disorder. Additionally, these environmental cues increase the oxidative status of plants by perturbing the delicate equilibrium between the production and the detoxification of reactive oxygen species (ROS) [1, 2]. As a response to the stressful conditions, plants induce the accumulation of compatible solutes like sucrose (Suc), fructose (Fru) and glucose (Glc) [3]. The accumulation of osmoprotectants maintains water retention in the plant cells without interrupting normal metabolism in a process called osmotic adjustment [4]. Osmotic adjustment sustains the cellular turgor for plant growth and survival during stressful environments and increases its capacity to detoxify ROS [3, 5]. Thus, the ability of plants to orchestrate carbon assimilation and sugar metabolism could determine plant survival during stressful environments [4, 6].

Primarily, carbohydrates generated by photosynthesis are either transiently stored as starch or reversed to Suc and transported to the heterotrophic organs [7]. Under environmental stresses, starch is converted to Suc as energy supply to support plant survival when photosynthesis becomes insufficient [8]. Therefore, Suc is an essential molecule that accumulated after short-term of abiotic stresses and it is positively correlated with abiotic stress tolerance of plants [9–11]. However, Suc cannot be used directly and its catabolism is necessary to be involved in abiotic stresses responsive pathways [12–14].

In plants, Suc can be catalyzed reversibly by the Suc synthase enzyme (SuSy, EC 2.4.1.13) in the presence of UDP to produce UPD-Glc and Frc in the cytoplasm, which provides substrates for starch and proteins biosynthesis [15]. Meanwhile, the invertase enzyme (INV, EC 3.2.1.26) irreversibly hydrolyzes Suc to form two hexoses (Glc and Frc) [16]. It has been reported that INV members can be clustered into acidic INVs (AINV) and structurally unrelated alkaline/neutral INVs (A/NINV) based on the similarity of protein sequence and the biochemical property of their pH optima [17, 18]. Furthermore, AINV proteins are disturbed into cell wall invertase (CWINV) and vacuole invertase (VINV) according to the protein subcellular localization [19, 20]; and A/NINV proteins are diverged into several distinct isoforms localized in cell membrane, cytoplasm, nucleus, chloroplast, and mitochondria [13, 20–22]. Unlike CWINVs and VINVs that act at acidic pH (4.5–5.5), A/NINVs function at optimal pH of 7.0–7.8 [23]. Both VINV and A/NINVs are soluble proteins with an acidic *pI*, while CWINVs are insoluble with basic *pI* to facilitate the interaction with the cell wall [23].

Physiological and genetic evidences indicated to the conserved functions of *CWINV* genes in plant growth, pollen fertility, oval formation, seed and fruit development, and pathogens defense through regulating the Suc unloading to sink tissues and controlling the sugar signals [19, 24, 25]. Besides, *VINV* isoforms were revealed to regulate hexoses accumulation and sucrose metabolism in fruit [26]. On contrary, several reports demonstrated that the *A/NINV*s play more prominent roles than the *AINV*s during stressful conditions [27]. For example, the *Arabidopsis thaliana AtA/NINVG* controls osmotic stress-induced developmental process [28]. Moreover, the wheat alkaline *Ta-A-Inv* was revealed to play a role in response to environmental stresses [29]. *Arabidopsis* mitochondrial *AtA/NINVC* and *AtA/NINVA* maintain the function of mitochondria and thus contribute in facilitating the energy demands and the ROS homeostasis for plant stress tolerance [30, 31]. In parallel, *Arabidopsis* chloroplastic *AtA/NINVE* regulates carbon balance between the cytosol and the plastids, affecting the function of photosynthetic apparatus [32, 33]. However, INV enzymes activities and gene expression profiles showed diverged manner of response in different plant tissue under various abiotic stresses, indicating that *INV* genes function distinguishably and un-redundantly during environmental stresses [27, 30, 31]. In consideration of their important roles, *INV* gene family have been previously reported in *Arabidopsis* [31, 34], rice [16], tomato [21], pepper [35], cassava [36, 37], sugarcane [27], and maize [38]. However, *INV* genes have not been identified and characterized systematically in *Citrus*, an economically important fruit crop cultivated worldwide.

Most of the *Citrus* cultivars do not have best combination between fruit qualitative and quantitative traits with environmental stresses tolerance traits. Thus, the genetic improvement for new *Citrus* varieties is necessary to overcome the barriers of production [39]. The biological nature of *Citrus* growth makes the conventional breeding programs incompetent to achieve the genetic improvement of *Citrus* [40–42]. Therefore, modern biotechnologies and genetic engineering are promising approaches to beat the disadvantages of traditional breeding programs [43]. In this regard, identifying genetic mechanisms involved in response to abiotic stresses becomes essential for the genetic improvement programs of *Citrus* [44].

Despite the advanced progress in the genome sequencing of *Citrus* species [43, 45, 46], genome-wide identification of *INV* gene family has not studied yet in *Citrus*. In this report, we performed genome-wide characterization and expression profiling analysis of INV family members from the cold-hardy *P. trifoliata* (the common rootstock of *Citrus*). Furthermore, we assessed the relationship between the accumulation of sugars and the INVs’ activity in the *Citrus* and its relative species. Consequently, our study lays a foundation for functional characterization of *INV* genes in abiotic stresses tolerance, especially for cold response in *Citrus*.

## Materials and methods

### Plant materials and growth conditions

To obtain insight about the freezing tolerance of *Citrus* species, six *Citrus* and its relative species (Ptr; *P. trifoliata,* Ci; *C. ichangensis*, Cs; *C. sinensis*, Cg; *C. grandis*, Cl, *C. limon* and Fj; *Fortunella japonica*) were harvested from the National Center of Citrus Breeding at Huazhong Agricultural University, Wuhan, China. The seedlings were grown in soil and placed in controlled chamber with the following conditions:14 h light/10 h dark cycle, constant temperature of 23 °C, 65% relative humidity, and uniform illumination of 120 μmol. m^− 2^.s^− 1^ (Unless otherwise stated, all plant growth conditions are the same).

For cold treatment, two month-old seedlings growing in soil were exposed to 4 °C for indicated times (0 h, 6 h, 12 h, 24 h, 48 h, 72 h), and then recovered at room temperature for a day. For salinity treatment, two month-old plants growing in soil were transferred to 200 mM NaCl solution for indicated times (0 h, 6 h, 12 h, 24 h, 48 h, 72 h), and then recovered at water for a day. For dehydration treatment, two month-old plants were extracted from soil and putted on filter paper at room temperature for indicated times (0 h, 0.5 h, 1 h, 2 h, 3 h, 5 h), and then transferred to water for recovery. Plant materials from determined time points were harvested and directly immersed in liquid N_2_ and preserved at − 80 °C for further analysis. *P. trifoliata* flowers of spring blossom and matured *P. trifoliata* fruits were collected from the National Center of Citrus Breeding at Huazhong Agricultural University. All assessments were performed with three biological replicates.

### RNA extraction and quantitative real-time PCR (qRT-PCR) analysis

Total RNA was extracted from plant materials using a Trizol reagent (Aidlab, China) and treated with DNase I (Thermo, USA) to eliminate the DNA contamination. The RevertAid Reverse transcriptase cDNA synthesis Kit (Thermo, USA) was used to synthesize the first strand of cDNA. AceQ qPCR SYBR Green Master Mix (Vazyme, China) was utilized according to the manufacturer’s instructions to perform qRT-PCR via Applied Biosystems® QuantStudio™ 7 Flex Real-Time PCR System (ABI, USA). The gene expression levels were determined using gene-specific primers that designed with the aid of the primer designing tool of NCBI (https://www.ncbi.nlm.nih.gov/tools/primer-blast/). While, *Actin* was used as an internal reference gene to normalize the gene expression (unless otherwise stated, all primers are listed in Supplementary Table 1). The specificity of primer pairs were verified by semi-quantitative reverse transcription polymerase chain reaction (RT-PCR) and by melting curves. The cycling program comprised an initial denaturation step at 94 °C for 5 min, followed by 45 cycles of 94 °C for 10 s and 60 °C for 30 s. The gene expression analysis for each treatment or each time point was performed in four technical replicates from three biological replicates and representative data are shown as the mean values ± SE. The relative expression profile was calculated using the 2^-∆∆CT^ method relatively to the initial treatment time (0 h) that set as 1. The absolute gene expression was applied to analyze the transcription of *INV* genes in different tissue types according to previous report [47].

### Identification and cloning of *INV* gene family members

To identify the *INV* family members in *P. trifoliata*, the sequence of *Arabidopsis INV* members were used as queries for BLAST searching against *C. sinensis* in phytozome v13 tool (https://phytozome-next.jgi.doe.gov/blast-search), and *C. sinensis* and *P. trifoliata* genomes in sweet orange annotation project datasets (http://citrus.hzau.edu.cn/cgi-bin/orange). The sequences annotated as INV members with zero *E*-value were selected for further analysis. *INV* genes were cloned from the synthesized cDNA according the manufacturer’s instruction of the 2X Phanta Max Master Mix Kit (Vazyme, China). The purified PCR products were cloned into the pTOPO vector using pTOPO-Blunt simple cloning kit (Aidlab, China) according to the manufacturer’s instruction, and then transformed into *Escherichia coli* (*E. coli*) competent cells DH5α. The transformed bacteria were grown on selective solid LB medium containing appropriate antibiotic for overnight. The positive colonies were confirmed by PCR and them sequenced by Wuhan AuGCT DNA-SYN CO.LTD (China). All of used primers were listed in Supplementary Table 1.

### Bioinformatics analysis

The sequences obtained by the cloning were used as queries to search for the full-length open reading frame (ORF) using the ORF finder from NCBI (https://www.ncbi.nlm.nih.gov/orffinder/). In addition, the theoretical isoelectric points (*pI*) and molecular weights (MW) of INV proteins were calculated using ProtParam tool (https://web.expasy.org/protparam/). Multiple protein sequence alignment was performed using Clustal Omega, and visualized by GeneDoc. Analysis of conserved domains was done by SMART tool (http://smart.embl-heidelberg.de/). To study the phylogenetic relationship, multiple alignment of putative INV proteins from *Arabidopsis thaliana*, *Oryza sativa*, *Vitis vinifera*, *Zea may*, *Malus domestica* [21, 48] was applied on MEGAX software to generate the phylogenetic tree using the neighbor-joining algorithm. Bootstraps with 1000 replicates for Poisson correction model were performed to assess node support. The putative subcellular localization of INV proteins was predicted using TargetP, ChloroP, Mitoprot, and Yloc tools [49–53]. The conserved motif of PtrINVs were identified by the Multiple EM for Motif Elicitation (MEME Suite 5.4.1) server under the default setting (https://meme-suite.org/meme/tools/meme). Gene structures accompanied with phylogenetic tree were built using the Gene Structure Display Server (GSDS) (http://gsds.gao-lab.org/index.php). Chromosomal location of INV genes was designed using TBtool [54]. *Cis*-acting elements were identified using the PlantCARE database (http://bioinformatics.psb.ugent.be/webtools/plantcare/html/).

### Measurement of INV, SuSy and HXK enzymes’ activity

The activities of INV enzymes were measured according to the previous reports with minor modification [55, 56]. Frozen samples were ground to a fine powder using a pre-cold mortar and pestle. Total proteins were extracted from 0.3 g of ground samples using 3 mL of pre-cooled extraction buffer (40 mM Tris-HCl pH 7.6, 1 mM EDTA, 3 mM MgCl_2_, 1 mM benzamidine, 0.1 mM PMSF, 24 μM NADP, 14 mM β-mercaptoethanol) on pre-cooled mortar until material was fully thawed (20–30 min). The homogenate was collected and centrifuged at 10,000×*g* and 4 °C for 10 min. Subsequently, the crude extracts of soluble proteins were re-centrifuged as above for 20 min to discard any contamination. The soluble crude was dialyzed against 20 mM potassium phosphate buffer (PBK) (pH 7.4) at 4 °C for 12 h to remove the soluble sugars. The solid pellet was rinsed three times with ddH_2_O for 10 min to remove soluble proteins. The washed pellet was re-suspended in 1 ml of the pre-cooled extraction buffer with high salt concentration (1 M NaCl), and shook gently at 4 °C for 12 h in dark. The homogenate was centrifuged twice as above for 10 min to extract the cell wall bound proteins. The cell wall crude was dialyzed against 20 mM PBK buffer (pH 7.4) at 4 °C for 12 h to remove the soluble sugars and NaCl. All dialyzed extracts were divided into small aliquots and were snap-frozen in liquid N_2_ and preserved at − 20 °C for further analysis.

The activity of INV enzymes was tested according to [55, 56] with slight modification. To evaluate A/NINV enzyme activity, 20 μl of dialyzed extracts of soluble protein was incubated at 37 °C for 1 h with 180 μl of reaction buffer (50 mM Bicine-KOH buffer, pH 7.6) containing 100 mM Suc. To assess VINV and CWINV enzymes activities, 20 μl of dialyzed extracts from supernatant or pellet fractions, respectively, were incubated at 37 °C for 1 h with 180 μl of reaction buffer (50 mM sodium acetate at pH 4.5) containing 100 mM Suc. Assays without reaction were utilized as control to remove the background. The INV activities were estimated by evaluating Glc content produced by Suc hydrolysis and presented as μmol Glc. mg protein^− 1^. min^− 1^. Twenty microliter of dialyzed extract from the supernatant was applied to determine hexokinase (HXK) and SuSy activities via suitable commercial kits according to the manufacturer’s instructions (Nanjing Jiancheng Bioengineering Institute, Nanjing, China).

### Measurement of biochemical and physiological parameters

Glc content was measured spectrophotometrically by detecting the absorbance change at 505 nm according to the kit’s instruction (Nanjing Jiancheng Bioengineering Institute, Nanjing, China). Suc and Frc levels were assessed by particular kits following the manufacturer’s instructions (Nanjing Jiancheng Bioengineering Institute, Nanjing, China). Total sugar contents were examined by an appropriate kit (BC2710, Solarbio, China). Glc-6-phosphate (G-6-P) was enzymaticly tested by using a specific kit (ab83426, Abcam, China). EL%, chlorophyll *a* fluorescence, *Fv/Fm* ratio, the histochemical staining of nitro blue tetrazolium (NBT) and 3, 3′-diaminobenzidine (DAB) were conducted according to previous reports [13, 57].

### Statistical analysis

Experiments were performed using completely randomized design. Statistical analyses were performed using Statistical Package for the Social Sciences (SPSS) software. The significant difference are analyzed using one-way Analysis of variance (ANOVA) method, and displayed as (*P < 0.05, **P < 0.01, ***P < 0.001). Error bars refer to ± SE (*n* = 3).

## Results

### Genome-wide mining identified fourteen *PtrINV* genes in *P. trifoliata*

To identify *PtrINV* genes in *P. trifoliata* genome, *Arabidopsis INV* genes were used as query to BLAST the reference genomes of several *Citrus* species deposited in sweet orange annotation project datasets. All of the redundant genes were removed after a similarity sequence comparison. Finally, 14 non-redundant *INV* genes were identified. Seven putative A/NINV isoforms (*PtrA/NINV1*–*7*), two putative VINV isoforms (*PtrVINV1*–*2*) and five putative CWINV isoforms (*PtrCWINV1*–*5*) were cloned from the cDNA of *P. trifoliata* (Table 1)*.* According to the sequence analysis, the full-length open reading frame (ORF) of seven *PtrA/NINV* members ranged from 1677 bp (*PtrA/NINV3*) to 2037 bp (*PtrA/NINV7*); with putative protein sequences within the range 558–678 amino acids (aa) and molecular weights (MW) within the range 63.7–76.7 kDa (Table 1). The multiple sequence alignment of A/NINV proteins showed that the C-terminal of A/NINV proteins has a highly conserved glycoside hydrolase 100 (glyco-hydro-100) domain (Fig. S1), indicating that the variable N-terminus could contribute in the variation of the subcellular localization [29]. Moreover, the high similarity in the protein sequence between PtrA/NINV1 and PtrA/NINV6, PtrA/NINV2 and PtrA/NINV7, and PtrA/NINV3 and PtrA/NINV4 indicates that they are should be paralogous pairs. For the *PtrAINV* genes, the ORF ranged from 1203 bp (*PtrCWINV3*) to 2070 bp (*PtrVINV1*), and the length of the putative protein ranged from 573 to 687 aa with MW ranged from 64.4 to 76.4 kDa (Table 1). The putative protein sequences of the PtrVINV1, PtrVINV2, PtrCWINV1, PtrCWINV2, PtrCWINV4, and PtrCWINV5 contain typical motifs of plant AINVs, including the Suc-binding motif (NDPNG), the transition-state stabilizer motif (RDP), and the cysteine catalytic motif (MWECV/PD) (Fig. S2) [26]. The sequence comparison of AINV proteins elucidated that the N-terminal of VINV proteins contains longer signal sequence compared to that of CWINVs (Fig. S2).

**Table 1:** The molecular properties of invertase genes in *P. trifoliata*

Generic Name^a^	Subcellular location	*C. sinensis* ^b^	*P. trifoliata* ^c^	*C. grandis* ^d^	ORF Length	Peptide Length	Exon	Intron	MW (KDa)	*pI*
*PtrA/NINV1*	chloroplast	*Cs3g07570/* *Cs3g07590*	*Pt8g006360*	*Cg8g017930*	1932	643	6	5	72.2	6.92
*PtrA/NINV2*	chloroplast and/or mitochondria	*Cs3g15410*	*Pt5g012350*	*Cg2g032800*	2034	677	6	5	76.7	6.13
*PtrA/NINV3*	cytoplasm	*Cs3g22270*	*Pt5g005640*	*Cg3g019340*	1677	558	4	3	63.7	6.07
*PtrA/NINV4*	cytoplasm	*Cs8g08880*	*PtUn018320/* *PtUn017360*	Not existed	1692	563	4	3	64.5	6.08
*PtrA/NINV5*	cytoplasm	*orange1.1 t00516*	*Pt3g033910*	*Cg5g040500*	1980	659	4	3	74.3	5.75
*PtrA/NINV6*	chloroplast	*orange1.1 t01536*	*Pt4g015870*	*Cg7g009870*	1953	650	6	5	73.2	6.01
*PtrA/NINV7*	chloroplast and/or mitochondria	*Cs9g01760*	*Pt9g000720*	*Cg9g000750*	2037	678	6	5	76.4	6.59
*PtrVINV1*	Vacuole	*Cs5g09220*	*Pt3g007900*	*Cg5g008630*	2070	687	7	6	76.4	5.77
*PtrVINV2*	Vacuole	*Cs9g14590*	*Pt9g017350*	*Cg9g024960*	1929	642	7	6	71.3	5.61
*PtrCWINV1*	Cell wall	*Cs1g18220*	*Pt7g013220*	*Cg1g009770*	1722	573	7	6	64.4	9.06
*PtrCWINV2*	Cell wall	*Cs1g18230*	*Pt7g013220*	*Cg1g009770*	1743	580	6	5	65.5	7.98
*PtrCWINV3*	–	*Cs1g18240*	*Pt7g013230*	*Cg1g009760*	1203	400	7	6	44.7	8.45
*PtrCWINV4*	Cell wall	*Cs5g18640* */Cs4g18349*	*Pt1g003850*	*Cg4g006840*	1740	579	7	6	65.5	5.24
*PtrCWINV5*	Cell wall	*Cs6g14340*	*Pt6g007660*	*Cg6g015210*	1734	577	7	6	65.6	9.06

^a^Gene ID with Ptr indicates to *P. trifoliata*. ^b^ Transcript ID retrieved from *C. sinensis* genome in the sweet orange annotation project database (http://citrus.hzau.edu.cn/orange/). ^c^ Transcript ID retrieved from *P. trifoliata* genome in the sweet orange annotation project database (http://citrus.hzau.edu.cn/orange/). ^d^ Transcript ID retrieved from *C. grandis* genome in the sweet orange annotation project database (http://citrus.hzau.edu.cn/orange/)

As we know, the protein localization is closely related its function. The prediction of subcellular localization of PtrINVs was conducted by using different database and tools, like Mitoprot, TargetP, ChloroP and YLoc tools [49–52]. Accordingly, PtrA/NINV3, PtrA/NINV4 and PtrA/NINV5 were estimated to be localized in the cytoplasm with no transit peptide (Table 1). PtrA/NINV1 and PtrA/NINV6 proteins were predicted to have chloroplast transit signal. Furthermore, PtrA/NINV2 and PtrA/NINV7 proteins were found to have chloroplast and mitochondria transit signals (Table 1) which was consistent with the chloroplast and mitochondria localization of PtrA/NINV7 in the previous work [13]. On the other hand, YLoc tool identified vacuole signal in the PtrVINV1 and PtrVINV2 proteins (Table 1). The dedicated Targetp server recognized secretory signals in the protein sequences of PtrCWINV1, PtrCWINV2, PtrCWINV4 and PtrCWINV5 (Table 1). However, PtrCWINV3 is non-functional protein and the prediction tool does not identify secretory signal in its amino acid sequence. The various locations of INV proteins in different cellular compartments indicate to the diverse functions of these proteins in sucrose catabolism and sugar signaling transduction.

### The phylogenetic relationship and gene structure of *PtrINV* family members

**Fig. 1:**
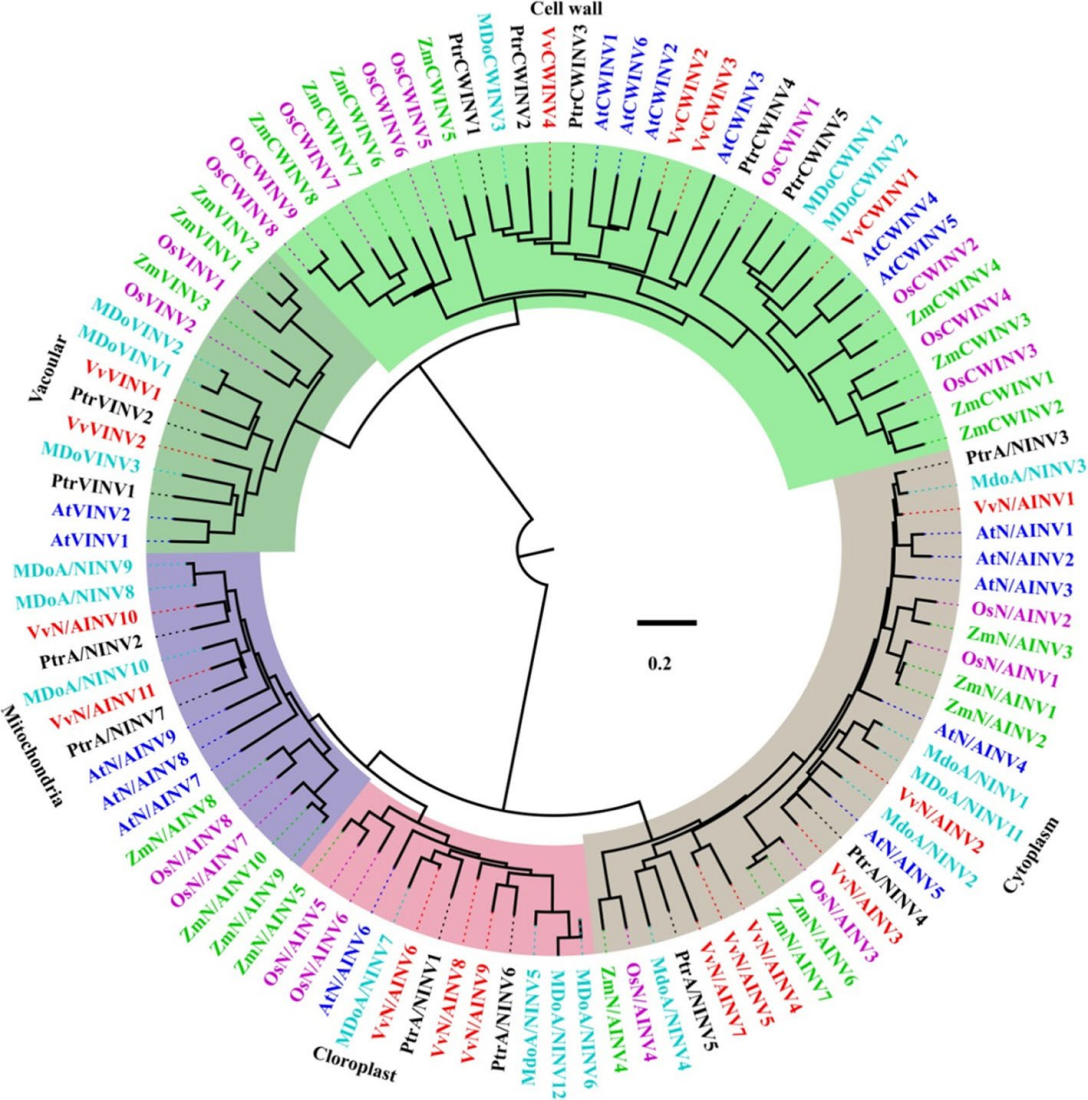
Phylogenetic analysis of INV proteins between different species. Phylogenetic tree was constructed with INV proteins of *Arabidopsis thaliana* (blue), *Oryza sativa* (purple), *Zea may* (green), *Vitis vinifera* (red), *Malus domestica* (cyno), and *P. trifoliata* (black). Multiple sequences alignment of INV proteins was conducted by MEGAX with the ClustalW method. The tree was constructed with the neighbor-joining method and bootstraps with 1000 replicates for poisson correction model. The dendrogram was categorized into five distinct subfamilies that highlighted using different colors. The light brown color refers to the cytoplasm localized INV proteins. The pink and purple colored clusters represent the chloroplast and mitochondria targeted INV isoforms, respectively. The acidic INV group was divided into cell wall bound INV peptides with light green color and vacuolar INV proteins with dark green color. The scale bar indicates to the relative amount of change along the branches. Accession number can be found in Supplementary Table S2

**Fig. 2:**
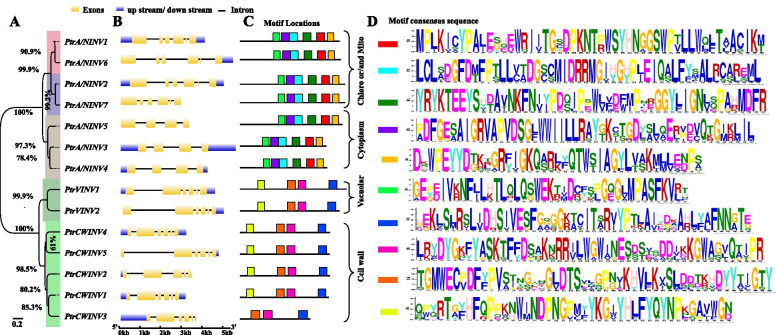
Phylogenetic relationship, gene structure and protein motifs distribuation of *PtrINV* family members in *P. trifoliata*. **A** The phylogenetic tree was constructed by MEGAX using the neighbor-joining algorithm and bootstraps with 1000 replicates for poisson correction model. The scale bar indicates to the relative amount of change along the branches, and numbers beside the branches of dendrogram represent bootstrap values. **B** Gene structure was built using the Gene Structure Display Server (GSDS). Brown rectangles refer to the exons, lines indicate to the introns, and blue rectangles show the upstream and downstream untransalated regions (UTR). **C** The distribution of protein conserved motif. **D** The sequence of motif

To classify the phylogenetic relationships of INV proteins, we constructed a phylogenetic tree based on 19, 21, 17, 17, 18 and 14 INV members from *Oryza sativa*, *Zea may* (monocots), *Arabidopsis thaliana* (dicot), *Vitis vinifera*, *Malus domestica* and *P. trifoliata* (fruit trees), respectively. The phylogenetic analyses showed that the INV family can be classified into two distinct groups (AINV and A/NINV enzymes), and each of these two groups can be further divided into multi-subgroups (Figs. 1 and 2). The phylogenetic tree elucidated a faster divergence in the AINV proteins in comparison with the A/NINV paralogs (Figs. 1 and 2). VINV proteins are branched off from CWINV paralogs during the evolution. Similarly, mitochondria and chloroplast localized INVs were divided from cytoplasmic INVs (Figs. 1 and 2). Interestingly, both AINV and A/NINV proteins of monocots, dicots and fruit trees were clustered into distinct clades (Fig. 1), suggesting that INV proteins were evolved after monocots, dicots and fruit trees divergence. Based on sequence alignment, PtrAINV proteins were classified into VINV (PtrVINV1 and PtrVINV2) and CWINV groups (PtrCWINV1–5) (Fig. 1). The PtrCWINV1, PtrCWINV2 and PtrCWINV3 isoforms are direct tandem repeats and clustered into one clade (Fig. 1). On the other hand, PtrA/NINV3, PtrA/NINV4 and PtrA/NINV5 were clustered into cytoplasm-localized clade (Fig. 1). Furthermore, PtrA/NINV1 and PtrA/NINV6 were diverged into chloroplast-targeted clade, and PtrA/NINV2 and PtrA/NINV7 were grouped into mitochondria-targeted branch (Fig. 1). Multiple sequence alignments and phylogenetic analysis revealed that INV proteins from *Oryza sativa*, *Zea may* (monocots), *Arabidopsis thaliana* (dicot), *Vitis vinifera*, *Malus domestica* and *P. trifoliata* shared high levels of similarity, which suggests similar functions for those homologous members.

For the gene structure analysis, the cytoplasmic localized *A/NINV* genes contain 3 introns and 4 exons, while the mitochondria and chloroplast localized *A/NINV* isoforms have 5 introns and 6 exons (Fig. 2B). Besides, all of *AINV* genes have 7 exons and 6 introns, except for 6 exons and 5 introns are identified in *PtrCWINV2* genomic sequence. Apart from the conserved gene structure, all *AINV* genes have the second small 9 bp exon (GATCCT/C/AAAT/C) that encodes DPN amino acid of the Suc-binding motif (NDPNG) (Fig. 2B) [26, 58]. For the protein conserved motif, 3 to 6 typical motifs were identified in INVs protein sequence. In general, members with close phylogenetic relationships had high sequence similarity and similar motifs (Fig. 2C-D). The presence of the same type of conserved motifs might indicate functional similarity among PtrINV family members.

### The chromosomal location of *PtrINV* genes in *P. trifoliata*

**Fig. 3:**
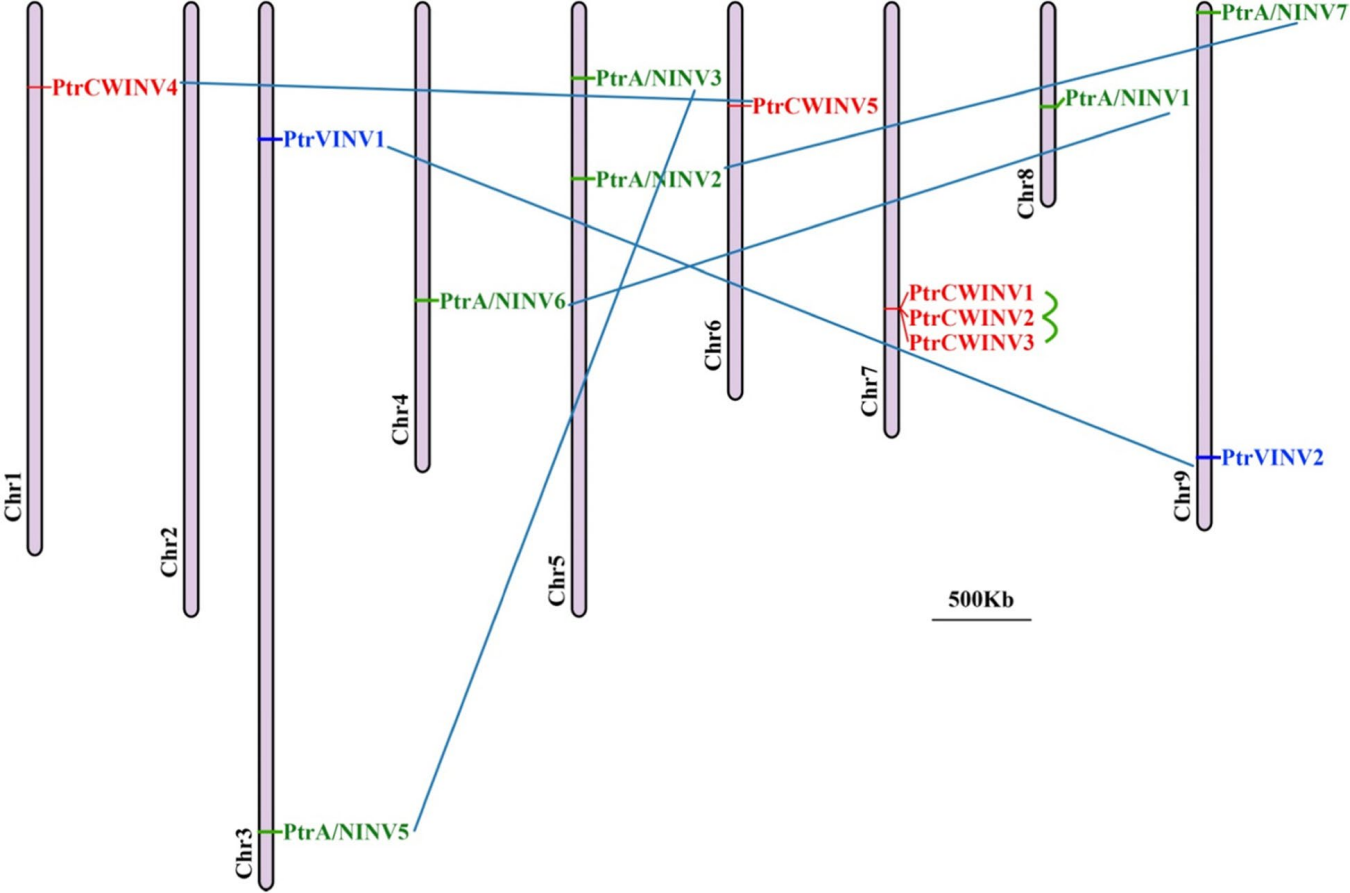
Chromosomal map of *PtrINV* genes within the genome of *P. trifoliata*. Thirteen *PtrINV* genes were mapped onto eight of nine *P. trifoliata* chromosomes (2n = 18). Chromosome numbers indicated below each chromosome. *PtrA/NINV* genes are shown in green, *PtrVINV* genes are in blue, and *PtrCWINV* genes are in red. Genome-wide or segmentally duplicated genes are linked by blue lines. Tandemly duplicated genes are linked with green lines. Scale bar is 500 k bases (kb)

Chromosomal location analysis showed that 13 *PtrINV* genes were distributed evenly on eight of the nine chromosomes (Chr) of *P. trifoliata* (Fig. 3), except for Chr 2. *PtrA/NINV4* was located on unattributed scaffold. *PtrA/NINV* genes were found on Chr 3, Chr 4, Chr 5, Chr 8 and Chr 9. Besides, our analysis showed that *PtrVINV1* and *PtrVINV2* are located on Chr 3 and Chr 9, respectively. Moreover, *PtrCWINV1*, *PtrCWINV2* and *PtrCWINV3* isoforms are direct tandem repeat on the Chr 7 of *P. trifoliata. PtrCWINV4* and *PtrCWINV5* are located on the Chr 1 and Chr 6, respectively.

Furthermore, we juxtaposed the distributions of *INV* genes within the genomes of *Citrus* species (*C. sinensis*, *C. grandis* and *P. trifoliata)*. Interestingly, the result demonstrated that *A/NINV1–3* genes were located on one chromosome (Chr 3) in *C. sinensis,* but they were found in two chromosomes (Chr 5, Chr 8) in *P. trifoliata*, and in three chromosomes (Chr 2, Chr 3, Chr 8) in *C. grandis* (Table 1). Moreover, *A/NINV4* was located on Chr 8 of *C. sinensis* and absent in the reference genome of *C. grandis* (Table 1). This diverse chromosome distribution pattern of *INV* genes between different *Citrus* species indicated that genetic variations was happened in the evolutionary process of the *Citrus*.

### Tissue-specific gene expression analysis of *PtrINV* genes in *P. trifoliata*

**Fig. 4:**
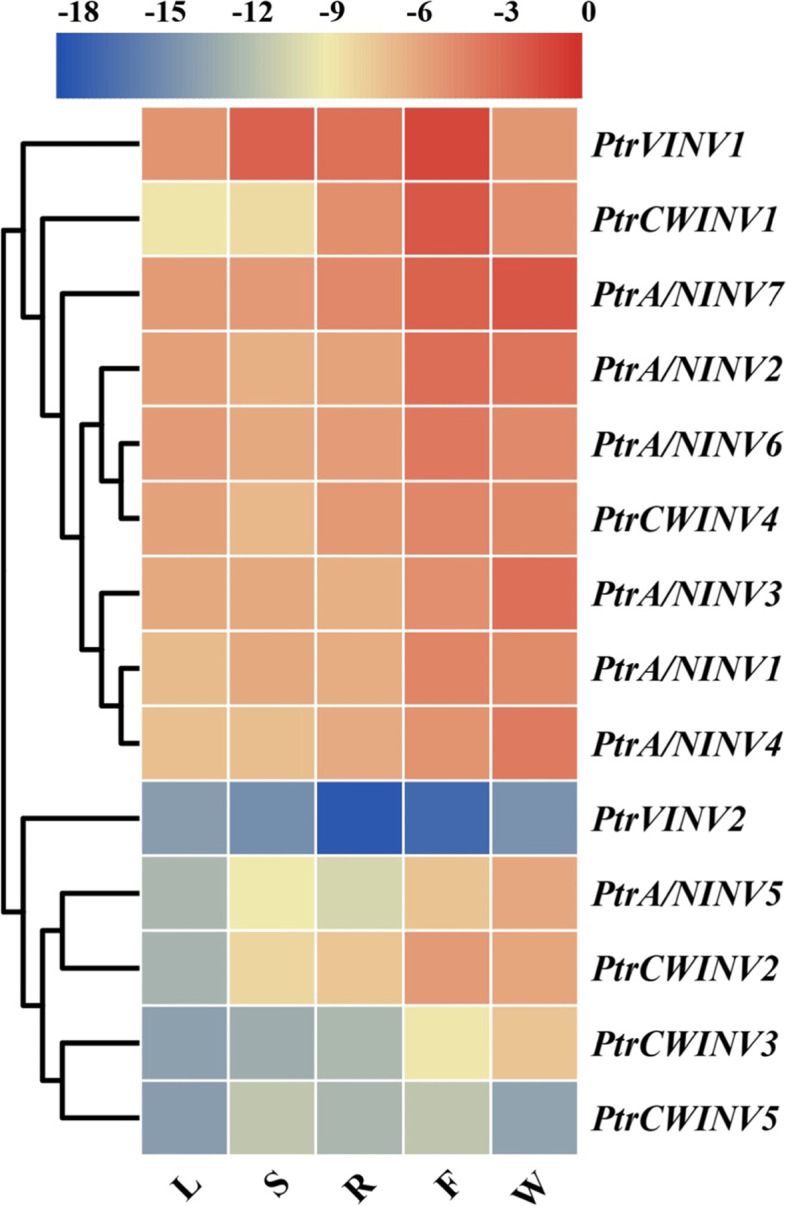
Tissue-specific expression profile of *PtrINV* genes in *P. trifoliata*. RNA was extracted from two-month old leaves (L), stems (S) and roots (R) of *P. trifoliata* seedlings, *P. trifoliata* flowers (W) of spring blossom, and matured *P. trifoliata* fruits (F). Real-time quantitative PCR (qRT-PCR) assay was performed using specific primers and *PtrActin* gene was utilized as reference gene. The transcript abundance was obtained based on standard curve using QuantStudio™ 7 Real-Time PCR Software v1.1 and normalization by the expression of *PtrActin*. Heatmap figure was constructed using Log_2_ of gene expression data by TBtools based on default features: euclidean distance method, and complete cluster method (*n* = 3). The red color indicates to highly expression levels, and blue color shows low expression levels. The cluster dendrogram is shown on left side of the heatmap

To investigate the biological role of *PtrINV* genes, the absolute quantification analysis was performed in different tissues according to the previous report [47]. Intriguingly, *PtrINV* genes were expressed in all tested tissues of *P. trifoliata* including leaves, stems, roots, fruits and flowers. In consistent with previous finding that some *PtrCWINV* genes were specifically expressed in sink tissues [59]. Our analysis exhibited that *PtrCWINV* genes were pronouncedly expressed in sink tissues (roots, fruits, and flowers) more than source tissues (leaves) (Fig. 4), suggesting the potential critical role of *PtrCWINV* genes in sink tissues [20]. Furthermore, *PtrA/NINV* genes with the exception of *PtrA/NINV5* were highly expressed in leaves and stems compared to the acidic *INV* genes with the exception of *PtrVINV1* and *PtrCWINV4* (Fig. 4), indicating that *PtrA/NINV*s and *PtrAINV*s could function diversely in different tissues. Moreover, *PtrA/NINV* genes were also expressed highly in flowers and matured fruits (Fig. 4). Among *PtrINV* family members, *PtrVINV2* and *PtrCWINV5* had lower transcript abundance in all tested *P. trifoliata* tissues. These differential expression patterns may provide important clues for exploring their functions in different tissues in the future.

### Gene expression analysis of *PtrINV* genes in response to osmotic stresses

**Fig. 5:**
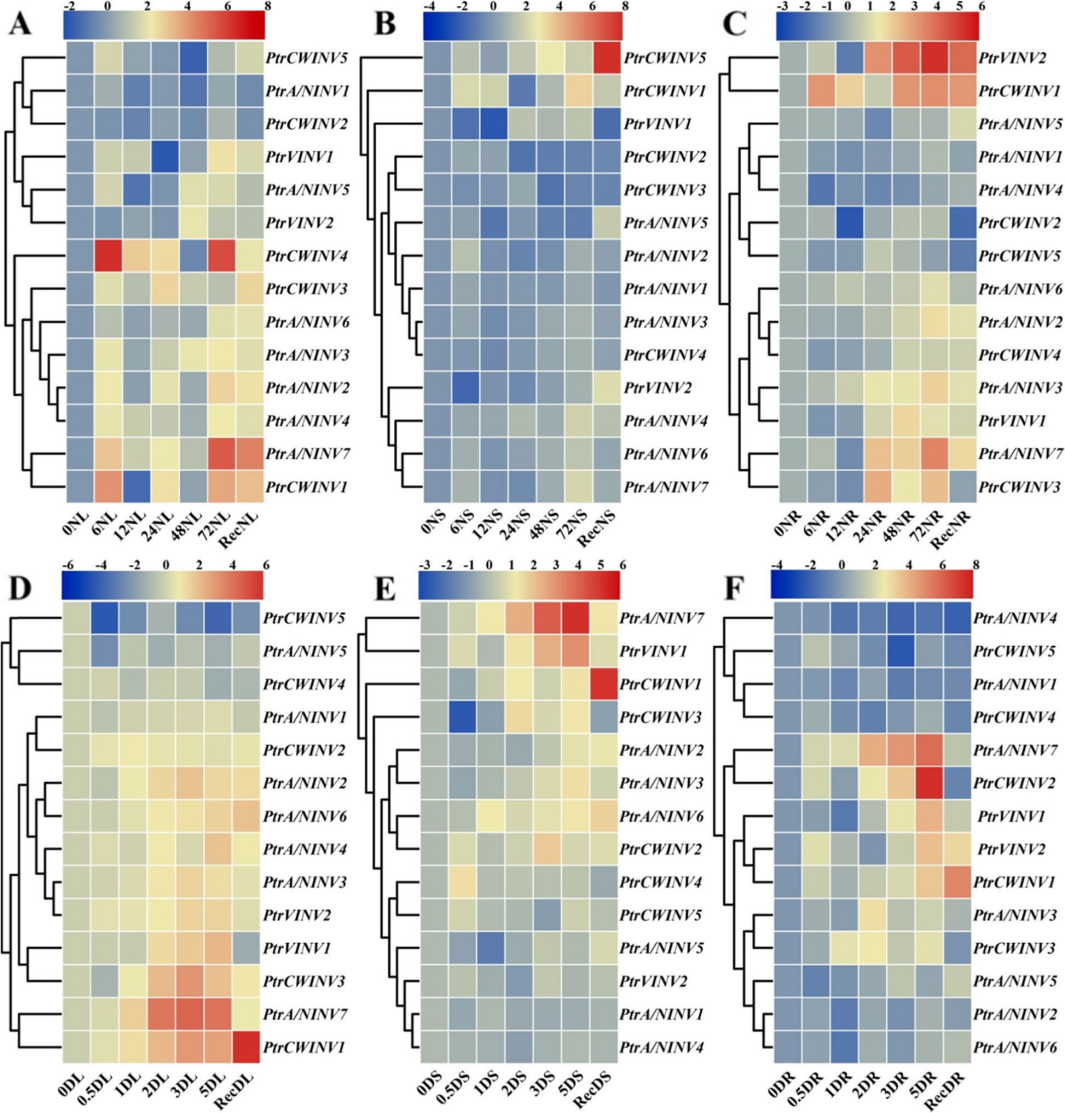
The relative transcript level of *PtrINV* genes in different tissue types of *P. trifoliata* during osmotic stresses. **A-C** The transcript profile of *PtrINV* genes in response to salt stress in leaves (**A**), stems (**B**) and roots (**C**) of two-month old *P. trifoliata* seedlings that exposed to 200 mM NaCl for indicated time (0 h, 6 h, 12 h, 24 h, 48 h and 72 h) and recovered for a day. (**D**-**F**) The transcript profile of *PtrINV* genes in response to dehydration stress in leaves (**D**), stems (**E**) and roots (**F**) of two-month old *P. trifoliata* seedlings that exposed to dehydration stress for indicated time (0 h, 0.5 h, 1 h, 2 h, 3 h and 5 h) and recovered a day. Real-time quantitative PCR (qRT-PCR) assay was performed using specific primers and *PtrActin* gene was utilized as a reference gene. The gene expression analysis for each time point was repeated for at least two times with four biological replicates, and representative data are shown. The relative gene expression was calculated using the 2^-∆∆CT^ method relative to the initial treatment time (0 h) that set as 1. The normalized values of the expression levels were converted into Log_2_ of expression values and visualized by heatmap figure. Heatmap figures were constructed by TBtools based on default features, Euclidean distance method, and complete cluster method (*n* = 3)

It was reported that sugars and INV activity have protective role against various stresses. To examine the contribution of *PtrINV* genes in response to abiotic stresses, gene expression analysis of *INV* family members was detected in salinity (200 mM) and dehydration treated leaves, stems and roots. Intriguingly, gene expression pattern showed that *PtrINV* genes were slightly induced in salt and dehydration treated stems (Fig. 5B and E). In general, *PtrA/NINV* genes tend to be induced in osmotic-treated leaves and *PtrAINV* genes were high expressed in osmotic-treated roots (Fig. 5A-F). This data refers to the putative roles of *PtrA/NINV* genes in source organs (such leaves), and the relevance of *PtrAINV* genes for sugar allocation to sink tissues (roots) in response to salt and dehydration stresses. The expression profile of *INV* genes in salt-treated leaves showed two distinct groups where *PtrA/NINV1*, *PtrA/NINV6*, *PtrVINV1*, *PtrCWINV2*, *PtrCWINV4* and *PtrCWINV5* were not induced (Fig. 5A). While, the expression levels of *PtrA/NINV7*, *PtrVINV2*, *PtrCWINV1* and *PtrCWINV3* were highly elevated in salt-treated roots (Fig. 5C). On the other hand, the transcript abundance of *PtrA/NINV1*, *PtrA/NINV5*, *PtrCWINV2*, *PtrCWINV4* and *PtrCWINV5* are slightly induced in dehydration-treated leaves (Fig. 5D). Furthermore, our data showed that *PtrA/NINV7* and *PtrCWINV1* are constitutively expressed in response to the osmotic stresses (salt and dehydration) (Fig. 5A-F). Moreover, *cis*-acting regulatory elements were analyzed in the promoter sequence of *INV* members. As shown in Fig. S3, MYB, WRKY and MYC transcription factors-binding sites, AT-rich motifs, light responsive-, stress responsive-, low temperature responsive- and phytohormones (such as gibberellin, abscisic acid, salicylic acid, and auxin) responsive elements were identified, suggesting that *INV* genes were undergone a complex transcriptional regulation in respond to various environmental conditions. Accordingly, these results revealed the potential function of *PtrA/NINV7* and *PtrCWINV1* in multiple abiotic stresses of *P. trifoliata*.

### Gene expression analysis of *PtrINV* genes in response to low temperature

**Fig. 6:**
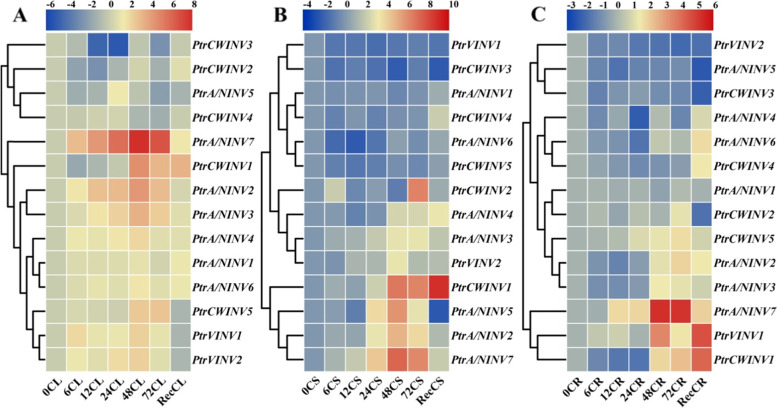
The relative expression patterns of *PtrINV* genes in different tissue types of *P. trifoliata* during low temperature. RNA was extracted from 4 °C treated leaves (CL, A), stems (CS, B) and roots (CR, C) of two-month old *P. trifoliata* seedlings for (0 h, 6 h, 12 h, 24 h, 48 h, 72 h) and recovered (Rec) at room temperature for a day. Real-time quantitative PCR (qRT-PCR) assay was performed using specific primers and *PtrActin* gene was utilized as a reference gene. The gene expression analysis for each time point was repeated for at least two times with four biological replicates and representative data are shown. The relative gene expression was calculated using the 2^-∆∆CT^ method relatively to the initial treatment time (0 h) that set as 1. The normalized values of the expression levels were converted into Log_2_ of expression values and visualized by heatmap figure. Heatmap figures were constructed by TBtools based on default features: Euclidean distance method and complete cluster method. (*n* = 3)

To test the contribution of *INV* genes in cold tolerance of *P. trifoliata*, we examined the gene expression of *INV* family members in three different tissue types (leaves, stems and roots) during cold stress (4 °C for 72 h). In cold-treated leaves, *PtrA/NINV5*, *PtrCWINV2*, *PtrCWINV3* and *PtrCWINV4* were reduced during low temperature, whereas other genes were induced under cold stress (Fig. 6A). On the other hand, the expression profile of *PtrINV* genes in cold-treated stems was divided into highly expressed genes (including *PtrA/NINV2*, *PtrA/NINV5*, *PtrA/NINV7* and *PtrCWINV1*) and slightly or not induced genes (Fig. 6B). Moreover, *PtrA/NINV7*, *PtrVINV1* and *PtrCWINV1* genes among other *PtrINV* genes are dramatically up-regulated in cold-treated roots (Fig. 6C). In general, our analysis manifested that *PtrINV* genes were more induced in cold-treated leaves compared to stems and roots. Furthermore, the transcript abundance of *PtrINV* genes with exception of *PtrA/NINV7* tends to down-regulate at the beginning of cold stress in stems and roots (Fig. 6A-C).

Intriguingly, the gene expression of *PtrA/NINV7* was expressed significantly after short-term (6 h) of cold stress and reach to highest level at 48 h in all tested tissue types compared to other *PtrINV* genes (Fig. 6A-C). Furthermore, the gene expression analysis of *PtrCWINV* genes in *P. trifoliata* manifested that *PtrCWINV1* among other *PtrCWINV* genes was predominantly up-regulated at late stage of cold stress in all tested tissue types. Consequently, our data revealed the potential importance role of *PtrA/NINV7* and *PtrCWINV1* for cold tolerance of *P. trifoliata*.

### Higher sugar contents and soluble invertases activities were correlated with the freezing tolerance of *Citrus* species

**Fig. 7:**
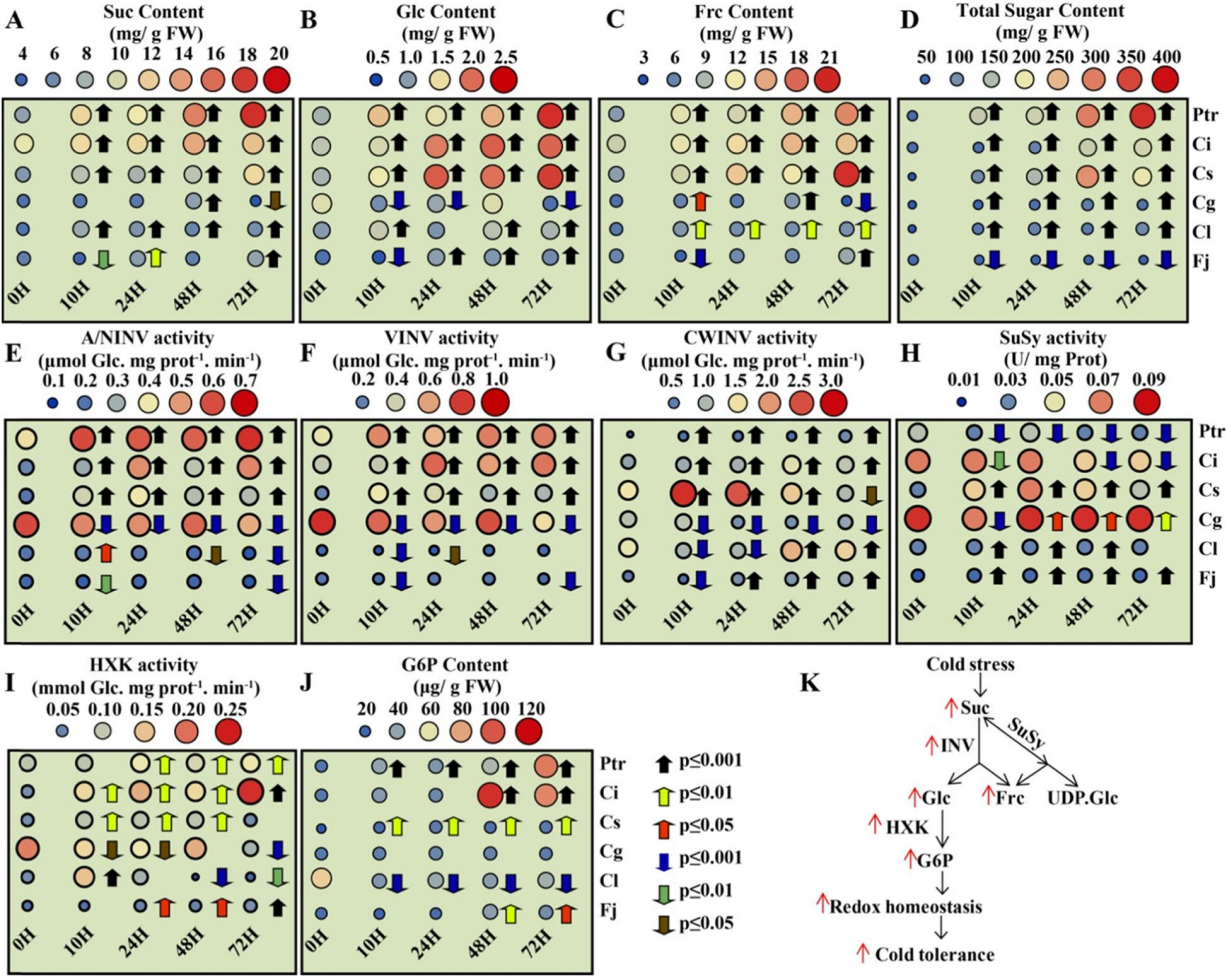
Comparison of sugars contents and activities of Suc hydrolyzing enzymes in *Citrus* and its relatives under cold stress. **A-D** Dynamic changes of Suc (**A**), Glc (**B**), Frc (**C**) and total sugar contents (**D**) during cold stress. **E**-**H** Dynamic changes in the activities of A/NINV (**E**), VINV (**F**), CWINV (**G**) and SuSy (**H**) enzymes during cold stress. **I**-**J** Dynamic changes of HXK activity (**I**) and G-6-P content (**J**) during low temperature. **K** Schematic representation displays the Suc catabolism pathways in response to cold stress. Ptr, *P. trifoliata;* Ci, *C. ichangensis*; Cs, *C. sinensis*; Cg, *C. grandis*; Cl, *C. limon* and Fj, *Fortunella japonica* seedlings were exposed to 4 °C for three days. The significant difference was analyzed using one-way ANOVA and displayed as (*P < 0.05, **P < 0.01, ***P < 0.001) and indicated by different colored arrow. Error bars refer to ± SE (*n* = 3)

To obtain insight about the freezing tolerance of *Citrus*, we selected six *Citrus* and its relative species for cold tolerance assay (Ptr, *P. trifoliata*; Ci, *Citrus ichangensis*; Cs, *C. sinensis*; Cg, *C. grandis*; Cl, *C. limon* and Fj, *Fortunella japonica*). Expectedly, *P. trifoliata*, *C. ichangensis* and *C. sinensis* displayed less freezing damage phenotype compared to other species, due to maintaining the integrity of photosynthetic apparatus and ROS homeostasis (Fig. S4). The dynamic changes in sugars contents were monitored after exposing two-month old seedlings to 4 °C in time-course manner (0 h, 10 h, 24 h, 48 h and 72 h). The results showed that Suc, Glc, and Frc contents were significantly accumulated in cold-tolerant species (*P. trifoliata*, *C. ichangensis* and *C. sinensis*) compared to that of sensitive ones (*C. grandis*, *C. limon* and *F. japonica*) after cold treatment (Fig. 7A-C). Moreover, total sugar content was significantly increased in all cold-treated leaves of species, except for *F. japonica*, which demonstrated the high accumulation of Suc, Glc, and Frc is essential for the cold-tolerance of *Citrus* species.

Suc is the final byproduct of photosynthesis and it have to be hydrolyzed before its involvement in downstream pathways. INV enzyme irreversibly hydrolyzes Suc to produce Glc and Frc, while SuSy reversibly catalyzes Suc to form UDP-Glc and Frc. However, which pathway is preferable for cold tolerance is unknown. INV activities (A/NINV, VINV and CWINV) in addition to SuSy activity in the cleavage direction were investigated during cold stress. As shown in Fig. 7E-F, the soluble invertases (A/NINV and VINV) were specifically activated in the freezing tolerant species in parallel with sugar accumulation during low temperature, revealing that higher accumulation of Glc and Frc in tolerant species might be caused by the soluble INVs activities. Furthermore, our data showed that CWINV activity was increased in *P. trifoliata, C. ichangensis*, *C. sinensis, C. limon* and *F. japonica* (Fig. 7G). Intriguingly, SuSy activity was significantly reduced during cold tress in the cold-tolerant species, particularly in the most cold-tolerant species (*P. trifoliata*). By contrast, SuSy activity was highly induced in the sensitive species (Fig. 7H), indicating that soluble INV-mediated Suc hydrolyzing is more favorable pathway for the cold tolerance of *Citrus* species.

It is well-known that Glc produced by INV activity undergoes phosphorylation with hexokinase (HXK) to enter glycolysis and pentose phosphate pathway, which are important pathways for ROS scavenging and energy supply [14, 31]. We hypothesized that if Glc and INV activity are positively correlated with cold tolerance, HXK activity and Glc-6- phosphate (G-6-P) content should take the same manner. To validate this notion, HXK activity and G-6-P content were also measured in the same extract crudes. In consistent with the sugar accumulation and INV activities, HXK activity and G-6-P content were noticeably elevated in the cold tolerant species compared to that of sensitive ones (Fig. 7I-J). Overall, our consequences emphasized that Suc, Glc and Frc contents were directly correlated with soluble INV activities in the cold tolerance of *Citrus* (Fig. 7K).

### The strong induction of *A/NINV7* was conserved in *Citrus* species during cold stress

**Fig. 8:**
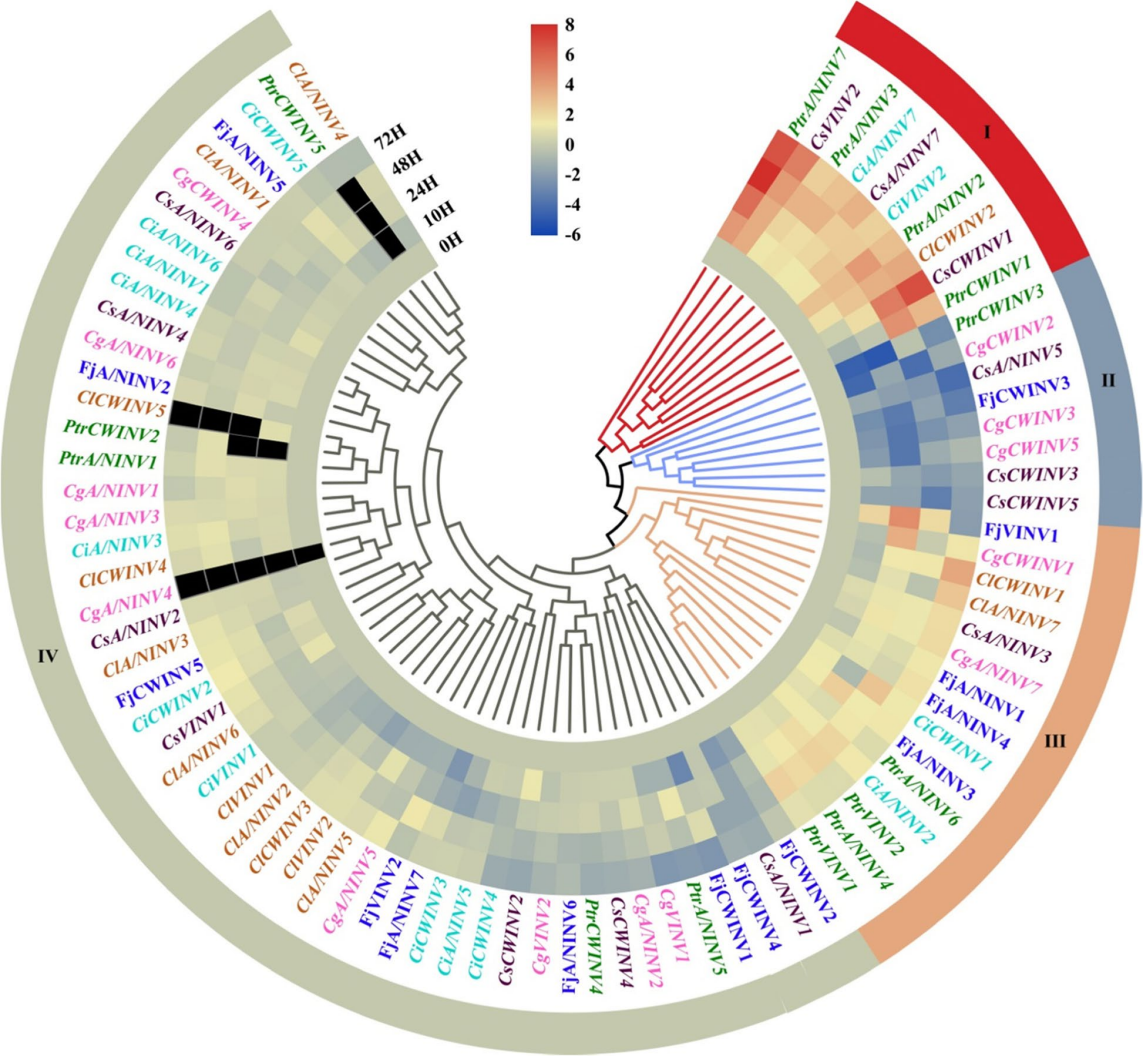
Relative expression level of *INV* family members during low temperature in *Citrus* or its relative species. Two-month old seedlings from six *Citrus* species were treated on 4 °C for indicated time (0 h, 10 h, 24 h, 48 h and 72 h) and the leaves were collected for further analysis. Ptr, *P. trifoliata;* Ci, *Citrus ichangensis*; Cs, *C. sinensis*; Cg, *C. grandis*; Cl, *C. limon* and Fj, *Fortunella japonica*. Real-time quantitative (qRT-PCR) assay was performed using specific primers and *PtrActin* gene was utilized as a reference gene. The expression profiles of *INV* genes under cold stress were classified into the highly expressed genes (I group), the moderately expressed genes (III group), the slightly expressed (IV group) and no induction or down regulated genes (II group), which were colored by red, brown, gray and blue, respectively. The gene expression analysis for each treatment or each time point was repeated for at least four times. The relative gene expression was calculated using the 2^-∆∆CT^ method relatively to the initial treatment time (0 h) that set as 1. The normalized values of the expression levels were converted into Log_2_ expression values and visualized by heatmap figure. Heatmap figures were constructed by TBtools based on default features, Euclidean distance method, and complete cluster method. Dark color refers to not determined values (*n* = 3)

To examine which *INV* isoform could correlate with the cold tolerance of *Citrus*, relative expression level of *INV* genes were determined in cold-treated seedlings of *P. trifoliata, C. ichangensis*, *C. sinensis*, *C. grandis*, *C. limon* and *F. japonica*. The expression profiles of *INV* genes under cold stress were classified into highly expressed, moderately expressed, slightly expressed, and no induction or down regulated groups (I, III, IV and II, respectively) (Fig. 8). Of note, we failed to record gene expression of *A/NINV4* from *C. grandis* during low temperature in qRT-PCR process (Fig. 8). Interestingly, the cold-hardy species (*P. trifoliata*) showed more induction of *PtrINV* genes including two cytoplasmic *A/NINV* genes (*PtrA/NINV3* and *PtrA/NINV4*), two chloroplast and mitochondria localized *A/NINV* genes (*PtrA/NINV2* and *PtrA/NINV7*), two *VINV* genes (*PtrVINV1* and *PtrVINV2*) and one *CWINV* gene (*PtrCWINV1*) (Fig. 8)*.* Contrary, the majority of *INV* genes were constant or repressed in the cold-sensitive *F. japonica* concomitant with the low accumulation of sugars during low temperatures (Figs. 7 and 8), implying to the central role of *PtrINV* genes in manipulating the sugar contents for cold tolerance of *Citrus*.

Among highly expressed genes, *A/NINV7* is strongly induced after short-term low temperature treatment in the cold tolerant species (*P. trifoliata, C. ichangensis*, *and C. sinensis*) (Fig. 8), which accompanied with increased A/NINV enzyme activity and significant accumulation of Glc and Frc during cold stress. Overall, *A/NINV7* was implied to be a potential conserved factor for the cold tolerance of *Citrus*.

## Discussion

In plants, invertase (INV, EC 3.2.1.26) enzymes catalyze Suc hydrolysis to produce Glc and Frc [60]. Ample evidences proofed that INVs are evolved to obtain diverse biochemical properties to fit Suc metabolism in multiple cellular compartments in response to varied conditions. Thus, INV enzymes are indispensable for plant growth, development and stress tolerance [13, 26, 31, 33, 61–63]. Various *INV* families were identified in previous reports, as the rice has 8 *A/NINV*s, 2 *VINV*s and 9 *CWINV*s [16], and *A. thaliana* contains 9 *A/NINV*s, 2 *VINV*s and 6 *CWINV*s [59]. Furthermore, 24 putative *INV* genes were identified in *Populus* genome, including 16 *A/NINV*s, 3 *VINV*s and 5 *CWINV*s [64]. Additionally, tomato also has 24 *INV* genes comprising 9 *CWINV*s, 2 cell membrane *INV*s, 11 chloroplast *INV*s, 1 cytosol *INV* and 1 vacuolar *INV* [21]. In fruit trees, peach genome contains 5 *CWINV* genes [26], and apple genome has 18 *INV* isoforms (3 *VINV*s, 3 *CWINV*s and 12 *A/NINV*s) [48]. Here, the genome-wide identification of *PtrINV* gene family in *P. trifoliata* showed less diversity compared to other species, as the genome of *P. trifoliata* has 14 non-redundant *INV* genes including 7 *A/NINV* genes, 2 *VINV* isoforms and 5 *CWINV* members (Table 1). This was consistent with the fact that *P. trifoliata* possesses less chromosome numbers and small genomic size [43, 45, 46]. The less abundance of *PtrINV* members in the *P. trifoliata* genome is presumably due to lower genomic duplication events that limit the expansion of the gene family.

Gene duplication ultimately culminates in producing proteins with sub-functionalization, neo-functionalization, or non-functionalization. In this report, genome-wide analysis and protein sequence comparison with functionally characterized proteins were performed to estimate the potential function of PtrINV proteins. The neighbor-joining phylogenetic trees constructed for PtrINVs with *Oryza sativa*, *Zea may*, *Arabidopsis thaliana*, *Vitis vinifera*, *Malus domestica* INVs demonstrate their evolutionary relationships and potential similarities in function (Fig. 1). These phylogenetic trees will be a useful reference for future studies on PtrINVs. Moreover, the motif analysis and characterization will also help in future exploration of gene functions. The conservation and divergence of motif numbers present in the PtrINV proteins (Fig. 2) are expected to lead to functional similarities or differences between various PtrINV family members.

Moreover, the bioinformatics analysis illustrated that the phylogenic relationship, the introns-exon structure, and the protein motifs distribution of *PtrINV* genes were strongly associated (Fig. 2). Gene structure and protein motifs distribution of *P. trifoliata A/NINV* and *AINV* genes were conserved and similar to *INV* genes from other plant species [38, 64], revealing the significance of this gene family for plant biology [59]. The protein sequence analysis showed that PtrAINVs and PtrA/NINVs did not share any common domain due to their independent evolutionary origin [59]. Accordingly, PtrAINV and PtrA/NINV proteins were found to possess different functional domains at their C-terminus including the glyco-hydro-32 domain and the glyco-hydro-100 domain, respectively. Moreover, the N-terminus of both subgroups are variable and have different signal peptides for several subcellular localization (Fig. S1 and Fig. S2). Furthermore, our observation exhibited that the protein sequence of PtrA/NINVs showed many subgroup-specific conserved residues in the glyco-hydro-100 domain (Fig. S1). However, the multiple sequence alignment of *P. trifoliata* AINV proteins revealed variation in the C-terminus localized cysteine catalytic motif (MWECV/PDF) at glyco-hydro-32 domain (Fig. S1 and Fig. S2) [26, 38]. This catalytic motif plays essential role for Suc hydrolysis and conformation of AINV proteins [59]. Interestingly, the PtrCWINV2 protein has the MWECVDF motif similar to other vacuolar isoforms (Fig. S2), suggesting that the variation in specific residues of this conserved motif could determine the substrate specificity and/ or the optimum pH [26, 64]. However, further work is needed to characterize the function of INV proteins with consideration of the protein structure.

The important functions of INVs in plant development and stress tolerance have been widely reported. Considering that the function of any given gene is closely related to its expression, we determined the expression patterns of PtrINVs in different tissues and in response to osmotic and cold treatments to evaluate their potential functions. As expected, the comprehensive expression analysis of *INV* genes in *P. trifoliata* that showed the variation of expression profiles of *INV* isoforms in response to multiple abiotic stresses and in different tissue types (Figs. 4, 5 and 6), suggesting that each member of *INV* gene family could play critical role in specific tissue during specific environmental condition [59]. Interestingly, the transcript levels of *A/NINV* genes were more abundant in leaves compared to that of the *AINV* genes in *P. trifoliata* (Fig. 4). Furthermore, the mRNA levels of *A/NINV*s were highly accumulated in leaves compared to that of stems and roots in response to cold, salinity and drought stresses (Figs. 5 and 6). Consistently, higher contents of Suc, Glc and Frc were directly correlated with higher activities of soluble INV enzymes during cold stress of *Citrus* species (Fig. 7). Besides, leaves of the cold-tolerant *Citrus* species exhibited less oxidative status and sustained higher efficiency of photosynthesis (Fig. S4). It is well-known that hexoses are not transported and they accumulated in the site of formation based on the cellular demand [12, 65]. Thus, the induction of *INV* genes during abiotic stresses is associated with high demand of hexoses in leaves to provide energy and osmoprotective substances. The byproducts of INV-derived Suc hydrolysis could be recruited for the protection of photosynthetic apparatus and ROS scavenging. This is more supported as several reports demonstrated that A/NINVs play more prominent role than AINVs during stressful conditions [27, 31]. Among *PtrINV* family members, *PtrA/NINV7* responded predominantly and was up-regulated to multiple abiotic stresses in leaves, stems and roots (Figs. 5 and 6), implying its potential central role for abiotic stress tolerance. Similarly, the strong induction of *A/NINV7* homologs in response to low temperature was conserved in the cold-tolerant *Citrus* species (Fig. 8). Thus, our insights indicate to the potential role of *A/NINV* genes for abiotic stresses tolerance in plant leaves.

## Conclusions

In the present report, we performed genome-wide identification, phylogenetic analysis, and spatiotemporal expression analysis of *INV* genes under multiple abiotic stresses in *Citrus* species. Fourteen INV members were identified in *P. trifoliata*. Furthermore, our insights demonstrated that INVs were involved in multiple abiotic stress response and INV enzyme pathway was preferable pathway for cold tolerance of *Citrus* accompanying with the increase of sugars content. Overall, these results provide a framework for understanding the potential physiological roles of INV members during abiotic stresses in *Citrus* species.

## Supplementary Information

**Additional file 1: Table S1.** List of primer sequences used in this study. **Figure S1.** Multiple sequence alignment of PtrA/NINV proteins. **Figure S2.** Multiple sequence alignment of PtrAINV proteins. **Figure S3.** *Cis*-acting regulatory elements analysis in the promoter of *PtrINV* genes. **Figure S4.** Freezing-tolerant *Citrus* species maintained the integrity of photosynthetic apparatus during freezing stress.

**Additional file 2: Table S2.** Accession numbers of all invertase genes from 5 plant species used in this article.

**Additional file 3.** The Ct values and qPCR’s raw data used to construct expression profiles of PtrINV genes in *P. trifoliata*.

## Data Availability

The data supporting the conclusions of this article are included within the article and its additional files.

## Abbreviations

A/NINV: Alkaline/neutral invertase
AINV: Acidic invertase
VINV: Vacuolar invertase
CWINV: Cell wall invertase
Suc: Sucrose
Glc: glucose
Fru: Fructose
ROS: Reactive oxygen species
