# Supplementary material for: Genome-wide identification and expression profiling of invertase gene family for abiotic stresses tolerance in Poncirus trifoliata
Source: BMC Plant Biol. 2021 Nov 25;21:559. doi: 10.1186/s12870-021-03337-3 (PMC8614057; doi:10.1186/s12870-021-03337-3)
Supplement: Supplementary file 1 — Additional file 1: Table S1. List of primer sequences used in this study. Figure S1. Multiple sequence alignment of PtrA/NINV proteins. Figure S2. Multiple sequence alignment of PtrAINV proteins. Figure S3. Cis-acting regulatory elements analysis in the promoter of PtrINV genes. Figure S4. Freezing-tolerant Citrus species maintained the integrity of photosynthetic apparatus during freezing stress. [file 12870_2021_3337_MOESM1_ESM.docx]

**Supplementary Table 1. List of primer sequences used in this study**

| Purpose | Primer name | Forward primer | Reverse primer |
| --- | --- | --- | --- |
| INV isolation | *Cs3g07570* | ATGACTGCTGCTGGGGAAGCAGTTC | TCATACTATGAAGGTCTGCTTTTTCCG |
|  | *Cs3g15410* | ATGAATTCAATTAATCTTCTGGG | TTAGACAAGAATTTGAGAC |
|  | *Cs3g22270* | ATGGATGGGACAAAAGAGG | TCAGCAATTCCAAGAAGATGATC |
|  | *Cs8g08880* | ATGGATACGCTGTGTACGGTAGCTGAG | TCAGAATGTCCATGAATGTGATCTTCTC |
|  | *orange1.1t00516* | ATGACAAAAAAGCTGCGAGATCTCTTACATG | TCAAAATGAGGCTGAGCGGCTGAGCC |
|  | *orange1.1t01536* | ATGGGAACTTCTGAGGCGGTTCTCC | TCAAACTATATAGGTCTGATTCAAATTC |
|  | *Cs5g09220* | ATCAAGCAGCAGCCCTCT | TTGGCACATCTTCTACAACTCT |
|  | *Cs9g14590* | CCCCATTTCACCATTCTC | TCGGCTCAGATGGATTTA |
|  | *Cs1g18220* | TCATCCAATACCGACCTT | CCAACTCCTCCACCCTTT |
|  | *Cs1g18230* | TACTGGTTCCACAAACAT | TCAACTAATTTGAGCCTT |
|  | *Cs1g18240* | ATGGTTAACTGGACACCA | TCAATTTATTTGAGCTTTC |
|  | *Cs5g18640* | ATGACATCTCCAGCTTTA | CTATAATTTAAAACTTTCT |
|  | *Cs6g14340* | CAAATGGCCCCATGTACT | TGAGTTCATCTTCACTGG |
|  | *Cs4g18340* | CCAATGTATTACAAGGGAG | ACACCAATGACATCCAAC |
| Gene expression | qRT.A/NINV1 | GGGGAAGCAGTTGTTCAGGT | CACCATGAAACGCACAGACC |
|  | qRT.A/NINV2 | CCCTTTTGTGGCAGTTCACG | GGAAGTGAGGAACCCAGCAA |
|  | qRT.A/NINV3 | TGCCAATTCGTGGTGGCTAT | GTTCCTCCCATCGTGCTTCA |
|  | qRT.A/NINV4 | TCTGTGGCTTCTCACTGCTG | CGGCAATTGACCAGGTTTGG |
|  | qRT.A/NINV5 | TAAAGCCGTGTCCGAGTGTC | AGCACCAGTACTCTCTCCGT |
|  | qRT.A/NINV6 | AGTGGCAGATCATCACAGGC | CTCAGCTACCTGTACCGCTC |
|  | qRT.A/NINV7 | GGGAGGTTTATAGGCAAGC | ACGGATCTGGGATCTTGC |
|  | qRT.VINV1 | ACAAAAACTGGTAGTAATCTTCTCC | GTCTAGCTCAAACTCGGCCA |
|  | qRT.VINV2 | GTTTGGGCTGCTAGTCAATGC | GCAGAAGTAAGTGTTCGTTCCTTTC |
|  | qRT.CWINV1 | GCTTTGTAGCCAAAAGGGCG | AGACCTGCTTTGGTCACTGC |
|  | qRT.CWINV2 | CCCGGTGGCTACTAACAGTC | ACTTCAATCCCGCATCACCA |
|  | qRT.CWINV3 | TGACCAAATTATCCAGGCTGAAC | CCTTTGGGCTCTTGGTGGAA |
|  | qRT.CWINV4 | CAATTAGTCCAGTGGCCCGT | GATCAACCCAAGCAGGGTCA |
|  | qRT.CWINV5 | GTCTCTTGCTGCCGTAAAGG | GTAGTACATGGGGCCATTTGGA |
|  | qRT.Actin | CCGACCGTATGAGCAAGGAAA | TTCCTGTGGACAATGGATGGA |

**
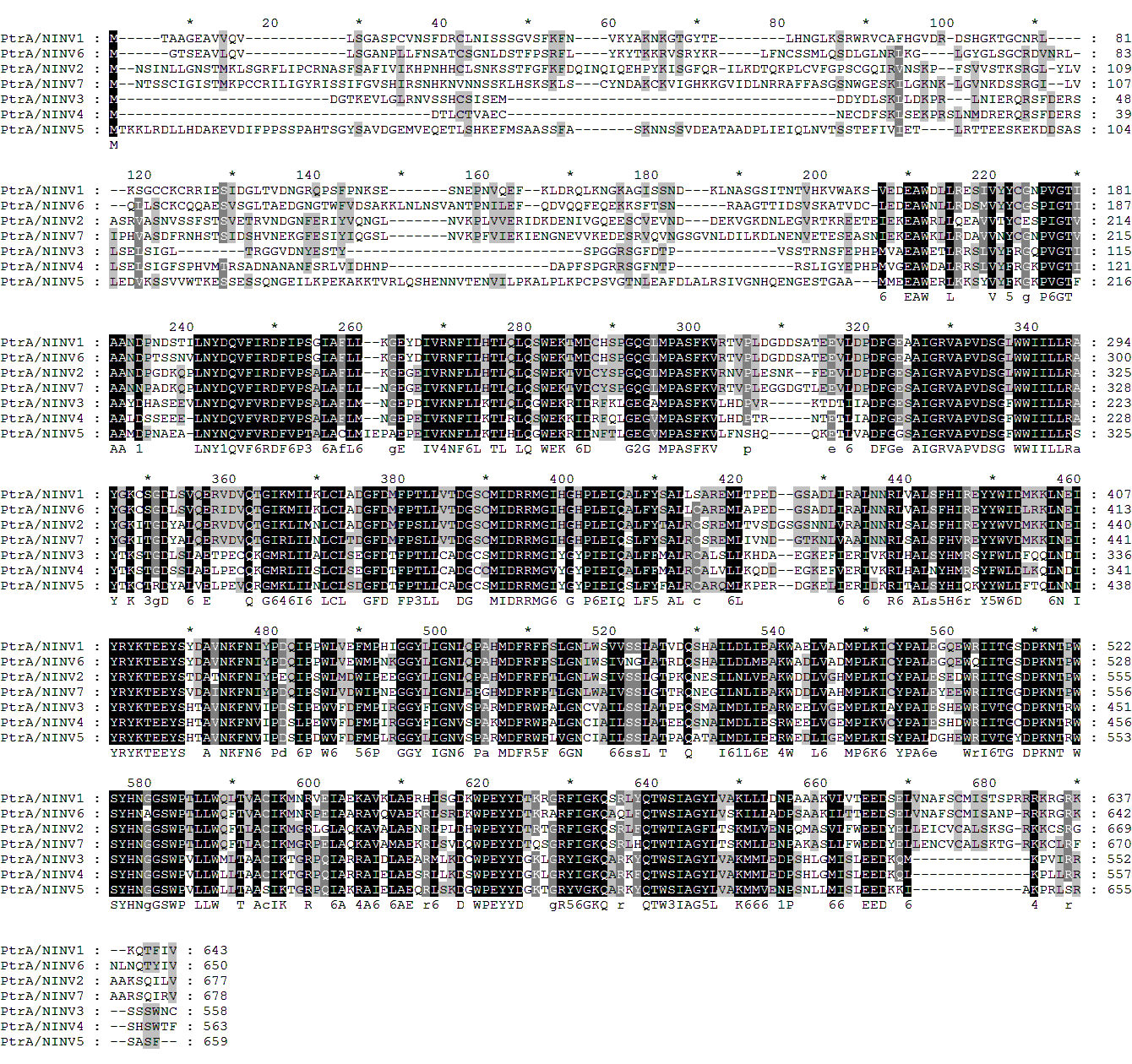
Fig. S1 Multiple sequence alignment of putative A/NINV proteins from *P. trifoliata*.** Dark and grey indicated residues refer to identical and highly conserved residues. The variable N-terminals of A/NINVs have signal peptides for different subcellular localization and the conserved C-terminal of A/NINVs possess the functional domain (glyco-hydro-100).


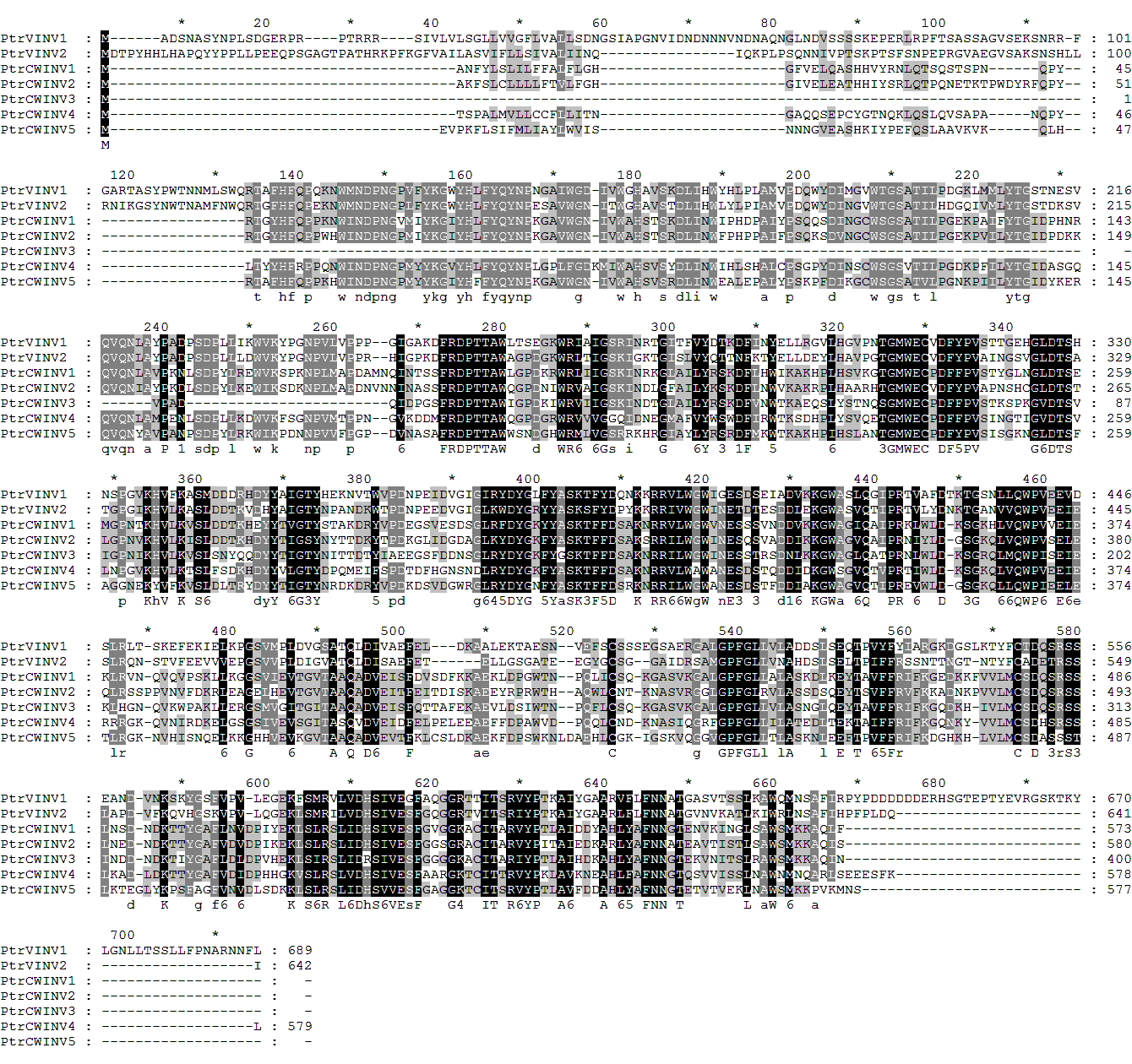


cysteine catalytic motif

Transition-state stabilizer

Sucrose-binding site

**Fig. S2 Multiple sequence Alignment of deduced AINV proteins from *P. trifoliata*.** Dark and grey residues indicate to identical and highly conserved residues. The variable N-terminals of the AINVs have signal peptides for different subcellular localization and the conserved C-terminal of AINVs possess the functional domain (glyco-hydro-32). The brown boxes showed the conserved motifs.


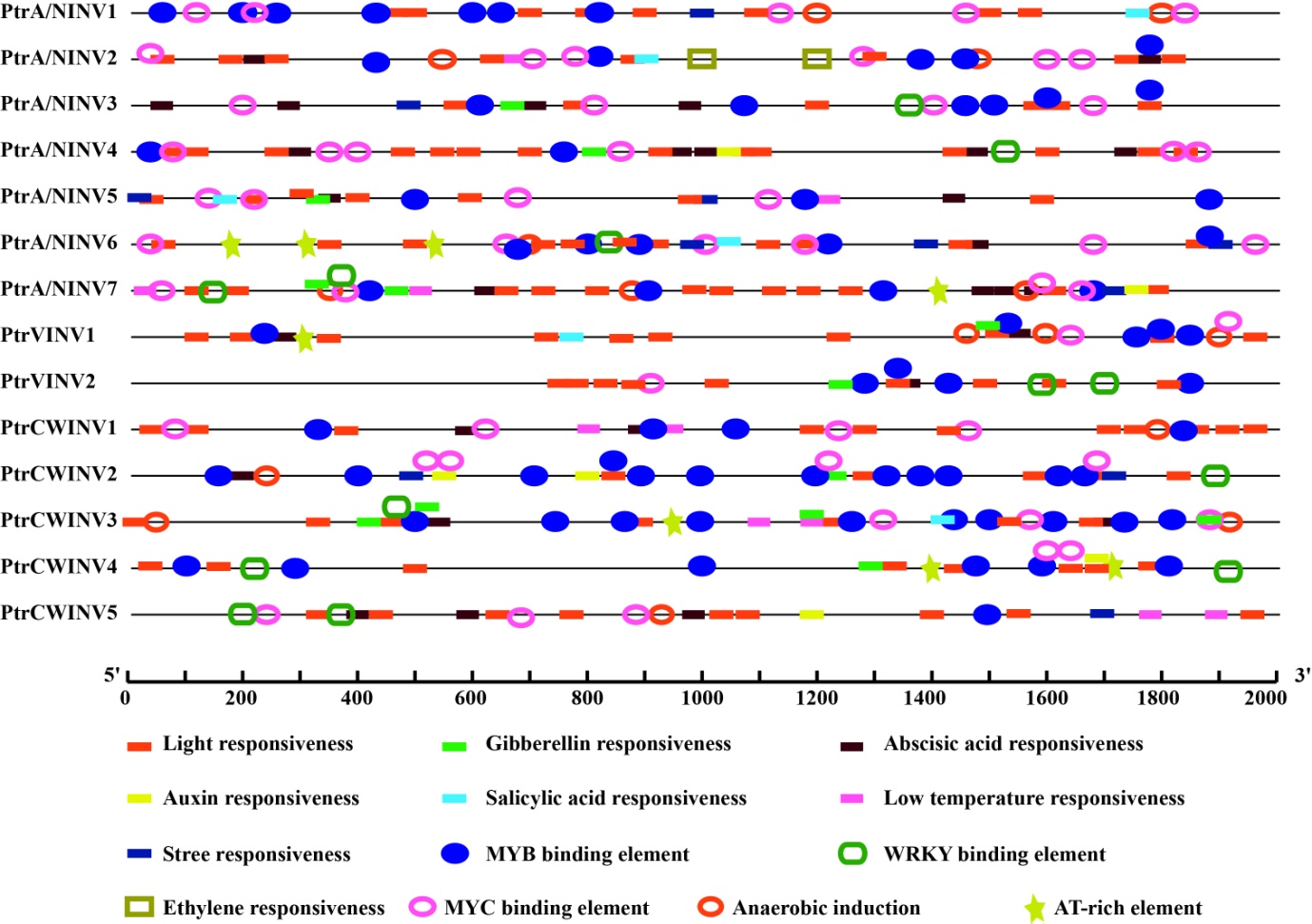


**Fig. S3 *Cis*-acting regulatory elements analysis in the promoters of *PtrINV* genes.**

*Cis*-acting regulatory elements were identified from 2 kb upstream of the start codon of the gene using the PlantCARE database. The promoter sequences were retrieved from the genomic sequence of *P. trifoliata* that deposited in the sweet orange annotation project database. Different shapes with different colors indicate to the distribution of the *cis*-elements on the promoters.


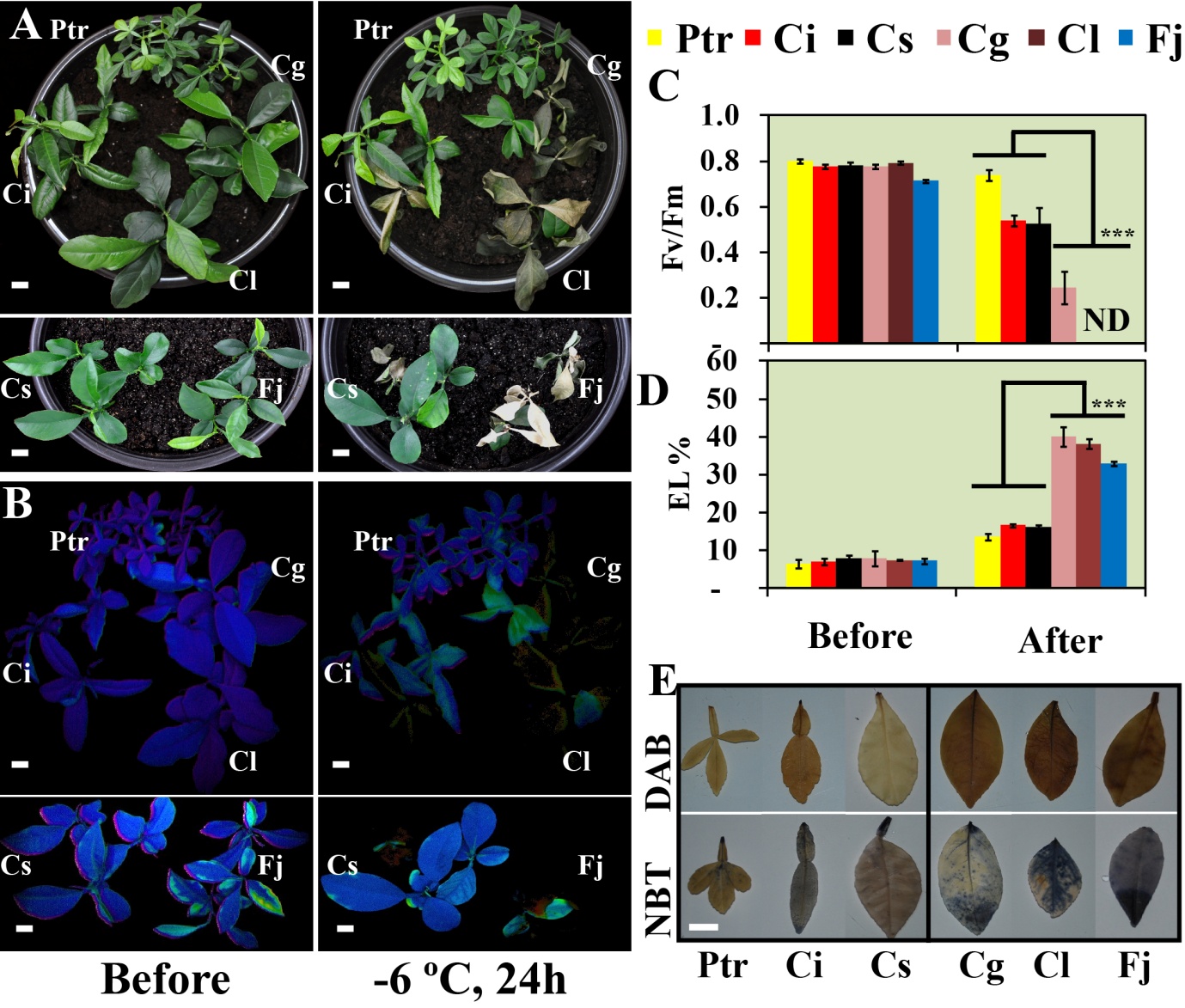


**Fig. S4 Freezing-tolerant *Citrus* species maintained the integrity of photosynthetic apparatus during freezing stress.**

**(A)** Two-month old seedlings of six *Citrus* or its relative species were acclimated on 4 °C for a day, then exposed to -6 °C for another day. Ptr, *Poncirus trifoliata*; Ci, *Citrus ichangensis*; Cs, *C. sinensis*; Cg, *C. grandis*; Cl, *C. limon* and Fj, *Fortunella* japonica.

**(B)** Chlorophyll fluorescence imaging, **(C)** *Fv/Fm* ratios, and **(D)** electrolyte leakage % (EL%) recorded before and after cold stress. The significant difference between the different species are analyzed using one-way ANOVA and displayed as (*P < 0.05, **P < 0.01, ***P < 0.001). Error bars refer to ± SE (n = 3).

**(E)** *In situ* histochemical staining of the 3, 3’-diaminobenzidine (DAB, upper panel) and nitro blue tetrazolium (NBT, down panel) show the accumulation of H_2_O_2_ and O_2_^·-^ radicals after freezing stress. Scale bar refers to 1 cm.
